# Neural computational model *GrowthEstimate*: A model for studying living resources through digestive efficiency

**DOI:** 10.1371/journal.pone.0216030

**Published:** 2019-08-28

**Authors:** Krisna Rungruangsak-Torrissen, Poramate Manoonpong

**Affiliations:** 1 Institute of Marine Research, Ecosystem Processes Research Group, Matredal, Norway; 2 Freelance Researcher, Bergen, Norway; 3 Embodied Artificial Intelligence and Neurorobotics Lab, Centre for Biorobotics, The Mærsk Mc-Kinney Møller Institute, University of Southern Denmark, Odense M, Denmark; 4 Bio-inspired Robotics and Neural Engineering Lab, School of Information Science and Technology, Vidyasirimedhi Institute of Science and Technology, Rayong, Thailand; University of Lincoln, UNITED KINGDOM

## Abstract

The neural computational model *GrowthEstimate* is introduced with focusing on new perspectives for the practical estimation of weight specific growth rate (SGR, % day^–1^). It is developed using recurrent neural networks of reservoir computing type, for estimating SGR based on the known data of three key biological factors relating to growth. These factors are: (1) weight (g) for specifying the age of the growth stage; (2) digestive efficiency through the pyloric caecal activity ratio of trypsin to chymotrypsin (T/C ratio) for specifying genetic differences in food utilization and growth potential, basically resulting from food consumption under variations in food quality and environmental conditions; and (3) protein growth efficiency through the condition factor (CF, 100 × g cm^–3^), as higher dietary protein level affecting higher skeletal growth (length) and resulting in lower CF. The computational model was trained using four datasets of different salmonids with size variations. It was evaluated with 15% of each dataset, resulting in an acceptable range of SGR outputs. Additional tests with different species indicated similarity between the estimated SGR outputs and the real SGR values, and the same ranking of wild population growth. The developed model *GrowthEstimate* is exceptionally useful for the precise and comparable growth estimation of living resources at individual levels, especially in natural ecosystems where the studied individuals, environmental conditions, food availability and consumption rates cannot be controlled. It is a revelation and will help to minimize uncertainty in wild stock assessment process. This will improve our knowledge in nutritional ecology, through the biochemical effects of climate change and environmental impact on the growth performance quality of aquatic living resources in the wild, as well as in aquaculture. The original *GrowthEstimate* software is available at GitHub repository (https://github.com/RungruangsakTorrissenManoonpong/GrowthEstimate). All other relevant data are within the paper. It will be improved for generality for future use, and required co-operations of the biodata collections of different species from different climate zones. Therefore, a co-operation will be available.

## Introduction

Growth estimation is very important for studying living resources, and precise estimation of the growth rate of organisms is important for minimizing uncertainty in stock assessment. Since food availability and environmental conditions influence the growth and distribution of animals in nature, the biochemical data involving food utilization and growth are essential. Different biochemical techniques have been uniquely developed over thirty years during Rungruangsak-Torrissen’s career for these purposes, through genetic variations in trypsin phenotypes affecting digestion and utilization of dietary protein, feed efficiency, maintenance rations, immunity, and growth in aquatic species [1–4]. Rungruangsak-Torrissen has recently summarized in details in her book about the developed analytical techniques for different biochemical factors, including the collection of biological samples [4]. These knowledges provide the concept for the development of the neural computational model *GrowthEstimate*.

In aquaculture, the weight specific growth rate (SGR, % day^–1^) is usually determined using body weights, at the start and at the end of a defined period of time, either according to [5] as:

$$SGR=100(e^{g}-1)$$ (1)

or according to [6] as:

$$SGR=100(ln\;W_{2}-lnW_{1})/(t_{2}-t_{1})$$ (2)

where *g* = (ln *W*_*2*_ *–*ln *W*_*1*_)/(*t*_*2*_–*t*_*1*_), *W*_*2*_ = Weight in g at day *t*_*2*_, *W*_*1*_ = Weight in g at day *t*_*1*_

These calculations are simple and very useful when studying the same individuals in a population, and when food availability and consumption rates are known within a defined period of time. However, in natural ecosystems where individual fishes, food availability and consumption rates cannot be controlled, other biological factors are needed for precisely estimating the growth of living resources. It is more important to study growth performance quality than merely growth performance, since it includes the level of protein deposition to indicate the quality of food for animal growth. The key biological factors, necessary to understand growth including genetics and suitable for the practical purposes of growth performance quality, have been studied intensively over three decades by Rungruangsak-Torrissen and her research team, by understanding a series of growth mechanisms through genetics, digestion and utilization of dietary protein, and effects of food and environments. These studies are summarized in [1–4]. The results indicate that dietary protein is the primary key nutrient for growth, regardless of eating habits (carnivores, omnivores, or herbivores), and the differences in the ability to digest the same dietary protein for utilization and deposition for optimal growth are genetically affected.

The growth and natural behavior of aquatic animals (variations in size, production levels, spatial distributions, and vertical movements) are dependent on feeding and optimal food utilization for optimal growth, whereas protein growth efficiency and maturation rate are the important central effectual mechanisms. Fish with a high somatic growth rate will stay in a suitable environment with food variety and a temperature suitable for optimizing food utilization and growth [1–4]. However, fish with a high maturation rate will show reduced somatic growth and use the energy for gamete development, probably through changing in vertical movement to a deeper sea level with colder temperatures [1–4]. Therefore, it is important for future research to study growth, maturation, and behavior through digestive efficiency and utilization of dietary protein, since these are sensitive to changes in both internal factors (genetics, age, and growth stage) and external factors (temperature, light, vaccine, and food quality and availability) [1–4]. The unique intensive studies on genetic variations in trypsin phenotypes have provided not only knowledge on the temperature effects on feed efficiency and growth [7], but also new knowledge on the temperature preferences for fish growth in nature [8]. In addition, growth is stimulated by an increase in trypsin specific activity and a reduction in chymotrypsin specific activity in the pyloric caeca and small intestine [9]. This has made the activity ratio of trypsin to chymotrypsin (T/C ratio) in the pyloric caeca and small intestine the key to digestive efficiency in growth potential, independent of the specific activity levels of the two proteases, and regardless of protein or lipid growth [10,11]. Moreover, the T/C ratio of the trypsin-like to chymotrypsin-like activity in the oocytes could also indicate oocyte growth for the female maturation rate, despite the lower specific activities of the two oocyte proteases caused by the lower oocyte protein level at a higher maturation rate [12].

The increase in growth is due to the deposition of both protein and lipid. In order to know whether the increased growth is due to an increase in the deposition of protein (protein growth) or lipid (lipid growth), the concentration ratio of protein to lipid (P/L ratio) in the white muscle must be studied. Otherwise, the study of the condition factor (CF, 100 × weight in gram × length in cm^–3^) could indicate protein or lipid growth. This is because a higher dietary protein level affected a higher P/L ratio in the body and white muscle, with a higher increase in skeletal growth (length) than weight (especially during a higher temperature season) resulting in lower CF [13]. Growth performance quality is dependent not only on weight, but also on the protein utilization and deposition affecting the CF values of living resources. However, protein utilization and growth are influenced by protein digestive efficiency (T/C ratio), whereas both trypsin and chymotrypsin specific activities are dependent on dietary protein levels [13] and food consumption rates [14]. With increasing nonlinear factors and unknown individual fishes, the estimation of SGR as nonlinear temporal data using conventional techniques with only the weight and time interval, commonly performed in aquaculture as in [5] and [6], becomes unsuitable. In fishery research, the importance of physiological and ecological processes affected by environmental drivers has been overviewed through available modeling approaches [15]. However, there are weaknesses in growth study models [16,17], and the stock assessment of fish populations requires a group of models to improve predictions due to the bias and uncertainty of individual model estimates [18]. Therefore, the aim of this study is to include other temporal data of the key biological factors, like weight (for specifying the age of the growth stage), T/C ratio (for specifying digestive efficiency and growth potential), and condition factor (for specifying the P/L ratio effect on growth), into a computational model to provide precise growth estimates of living resources. This is especially important for studies in natural marine and freshwater ecosystems where the individual fishes, food availability, and the consumption rate cannot be controlled. In addition, providing inputs with the different temporally biological data of weight, T/C ratio, and CF into one model could achieve a more effective solution for accuracy and certainty in estimating growth of living resources in the wild. No assumptions are needed when these biological factors are included in a model, because they represent the actual responses of the animals themselves. The biochemical techniques developed for studying the growth performance quality, and the collection of biological samples, are summarized in [4]. Most researchers are not yet aware of the unique knowledge of the T/C ratio for digestive efficiency relating to growth potential, and the CF for protein growth efficiency.

Among other techniques, recurrent neural networks (RNNs) of reservoir computing type, known as universal computations [19,20], appear appropriate for estimating (nonlinear) temporal or history-based data [21]. This kind of recurrent neural network consists of three layers (input, hidden, and output). The input layer receives input data and feeds it forward to the output layer through the reservoir-based hidden layer. The hidden layer has sparsely recurrent connections which are randomly assigned. Based on this setup, the hidden layer exhibits intrinsic dynamics and temporal memory, making the recurrent neural network suitable for learning and predicting temporal sequences. Only the connection weights in the output layer are trained while those of the input and hidden layers remain fixed. Therefore, training this network is much simpler than other types of RNNs. Accordingly, the reservoir-based RNN was used to develop our computational model to obtain precise growth estimates of living resources from weight, T/C ratio, CF, and their combinations. The original data from previous publications on different species at different age and growth stages, with variations in feeding and environmental conditions [9,11–13], are used to develop the neural computational model *GrowthEstimate*.

## Materials and methods

### Biological data for developing the model

**Fig 1:**
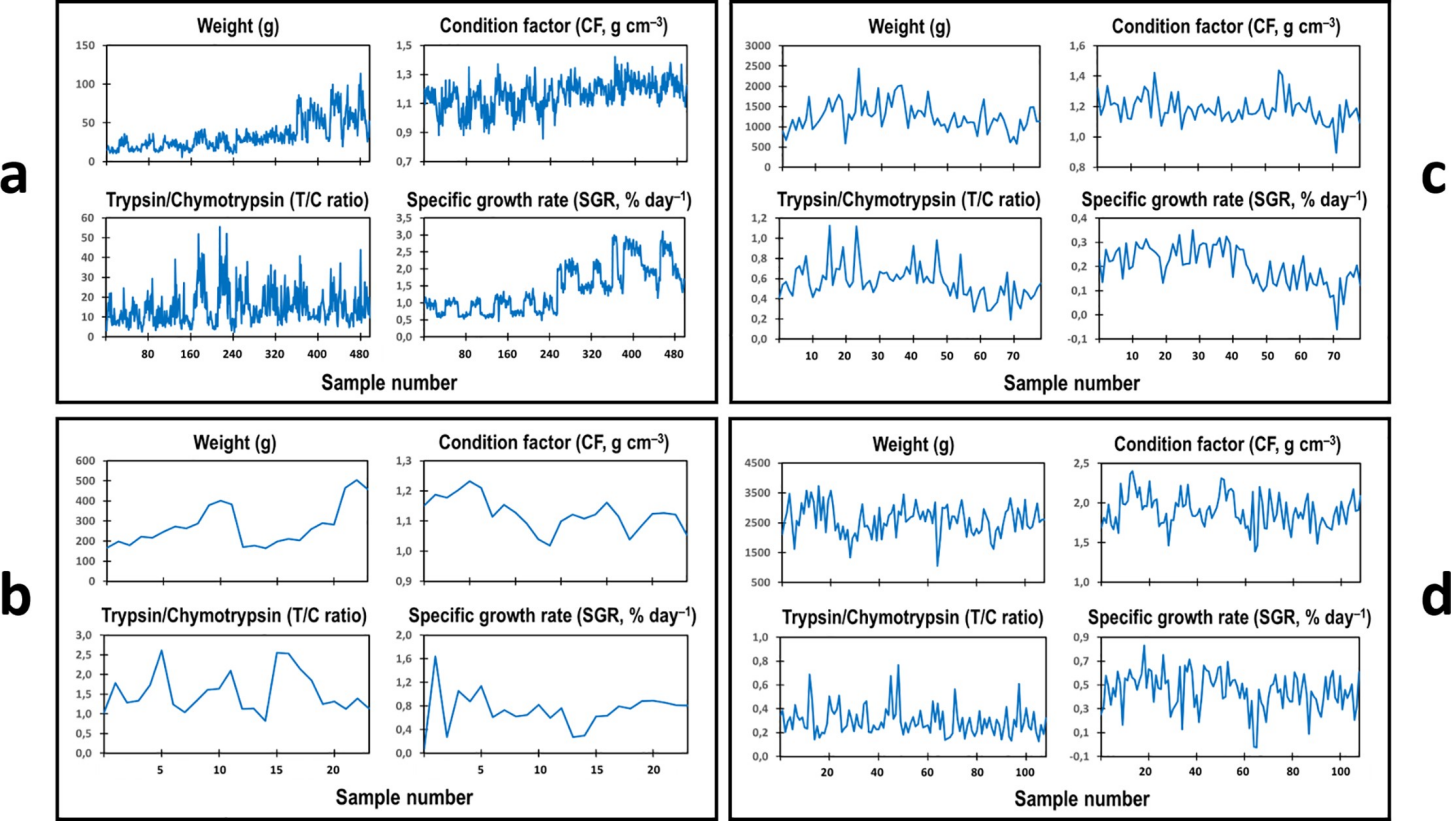
Datasets with weight, pyloric caecal T/C ratio, CF, and SGR. (a) Data1: Atlantic salmon at juvenile stage with 499 data points (original data from [9]). (b) Data2: Atlantic salmon at post-smolt stage with 24 data points (original data from [11]). (c) Data3: Atlantic salmon at adult stage with 79 data points (original data from [10]). (d) Data4: Rainbow trout at adult stage during maturation with 109 data points (original data from [13]). Weight, T/C ratio, CF, and SGR values will be normalized to a range of between –1 and +1 for training and testing our models.

The neural computational model *GrowthEstimate* for estimating the weight specific growth rate (SGR, % day^–1^) of living resources has been developed for generality. The original data from different species at different age and growth stages, and reared under different conditions, were used. Three inputs of known biological factors of weight (g), digestive efficiency (T/C ratio in the pyloric caeca and small intestine), and protein growth efficiency through the condition factor (CF, 100 × g cm^–3^), as previously mentioned, were exploited to obtain precise estimates of the SGR for individual fishes. The complete datasets with weight, T/C ratio, CF, and SGR of individual fishes were used for training the model. These were from Atlantic salmon (*Salmo salar* L.) at juvenile stage (Data1: *n* = 499, Fig 1A) [9], post-smolt stage (Data2: *n* = 24, Fig 1B) [11], and adult stage (Data3: *n* = 79, Fig 1C) [10], and from rainbow trout (*Oncorhynchus mykiss* Walbaum) at adult stage during maturation (Data4: *n* = 109, Fig 1D) [13]. The fishes were cultured with different feed quality or under different environmental conditions (temperatures or light regimes). Fish weight and length were measured at a specific time interval, and the pyloric caecae were dissected after the fishes were immediately killed by a blow to the head. The SGR was calculated according to Eq 1 [5]. The protease activities of trypsin (T) and chymotrypsin (C) in the pyloric caecal crude enzyme extract were determined using the specific enzyme substrates *N*-benzoyl-*L*-arginine-*p*-nitroanilide and *N*-succinyl-Ala-Ala-Pro-Phe-*p*-nitroanilide, respectively, and the increased absorbance per minute of the reaction product nitroaniline was measured spectrophotometrically at 410 nm [4,9–11,13]. The increase rate (per min) of nitroaniline level was obtained through the increase rate in its absorbance using a standard curve of *p*-nitroaniline. The enzyme activities were calculated in relation to the protein level in the pyloric caecal crude enzyme extract, and the enzyme specific activities of trypsin and chymotrypsin were expressed as μmol *p*-nitroaniline produced h^–1^ mg protein^–1^. The T/C ratio values were calculated. These data were used to develop the computational model for SGR estimation.

### Biological data for testing the model

**Fig 2:**
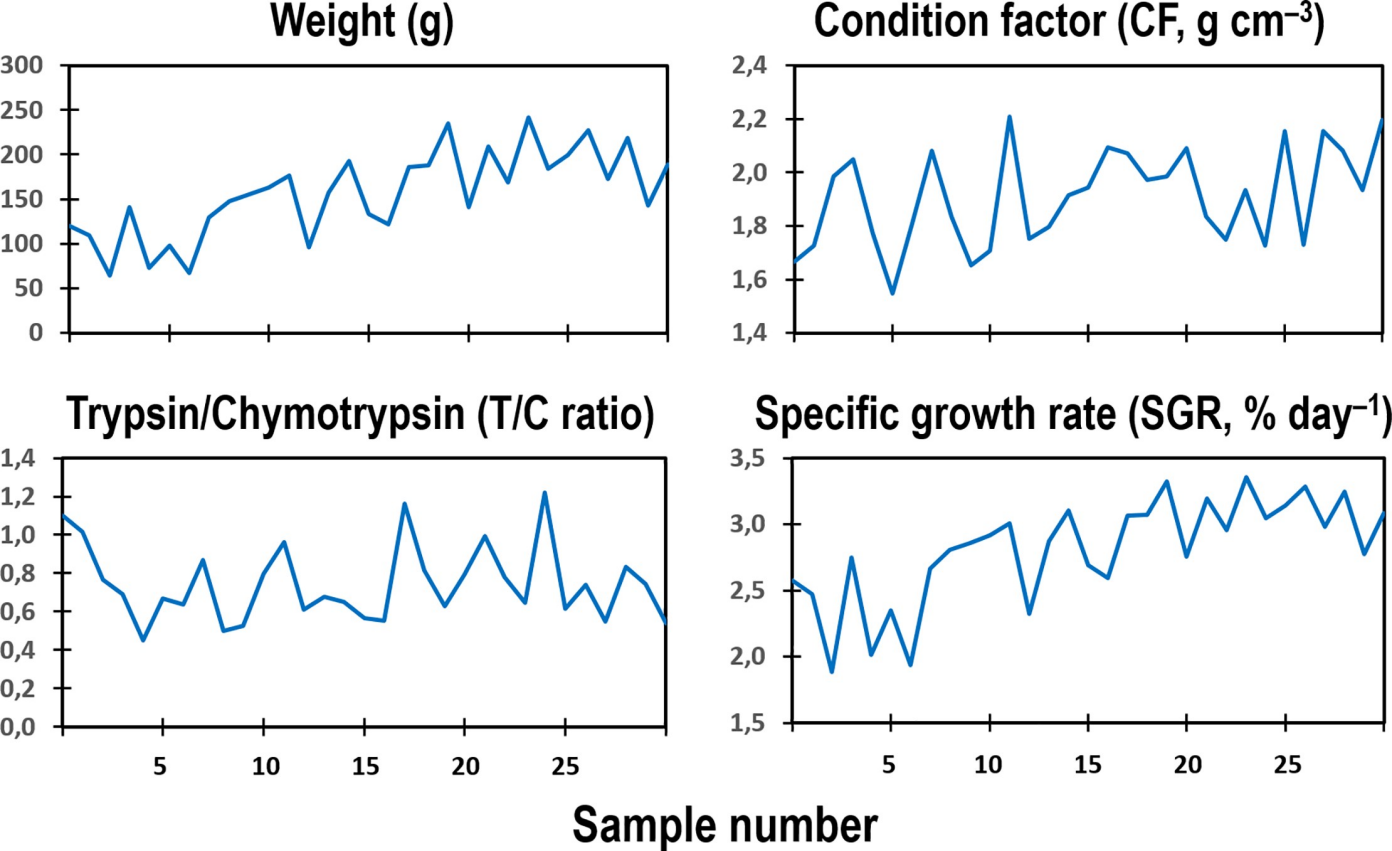
Dataset with weight, pyloric caecal T/C ratio, CF, and SGR. Data5 of Nile tilapia at adult stage with 31 data points. Weight, T/C ratio, CF, and SGR values will be normalized to a range of between –1 and +1 for training and testing our models. (original data personally provided by Karun Thongprajukaew, Prince of Songkla University, Thailand).

**Fig 3:**
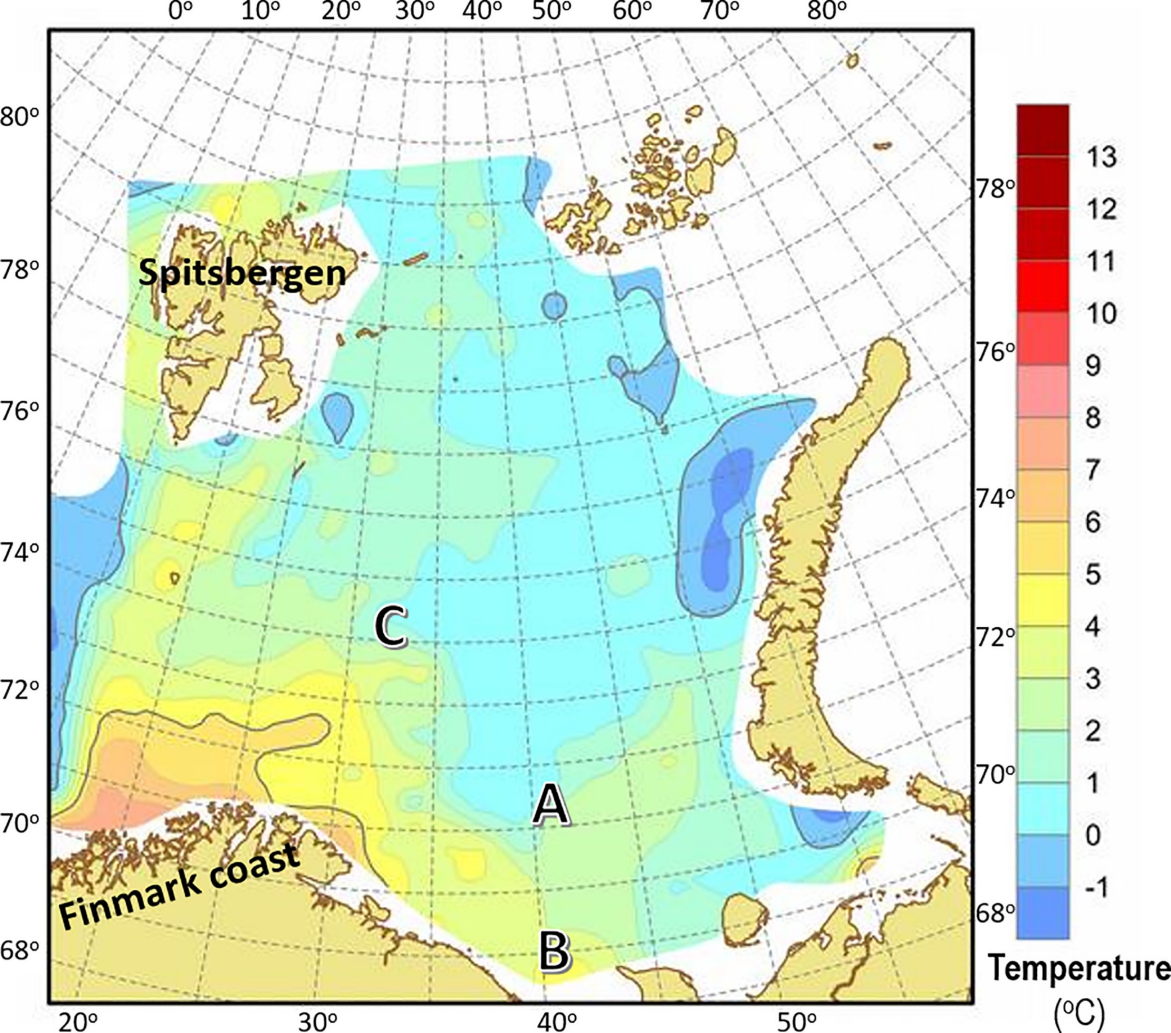
Distribution areas of wild Northeast Arctic cod in the Barents Sea. The three main studied areas (A, B, and C) and different bottom temperatures (^o^C) are illustrated. Area A was between North Kanin Bank and Eastern Basin, area B was Kanin Bank, and area C was Central Bank, with average bottom temperatures of 1.61 ^o^C, 3.45 ^o^C, and 1.69 ^o^C, respectively. The dataset of individual weights, pyloric caecal activity ratio of trypsin to chymotrypsin (T/C ratio), and condition factor (CF) of the wild Northeast Arctic cod was available [12]. Latitudes and longitudes are indicated on the frame. The picture was obtained from the routine cruise survey conducted by the Institute of Marine Research, Norway.

**Fig 4:**
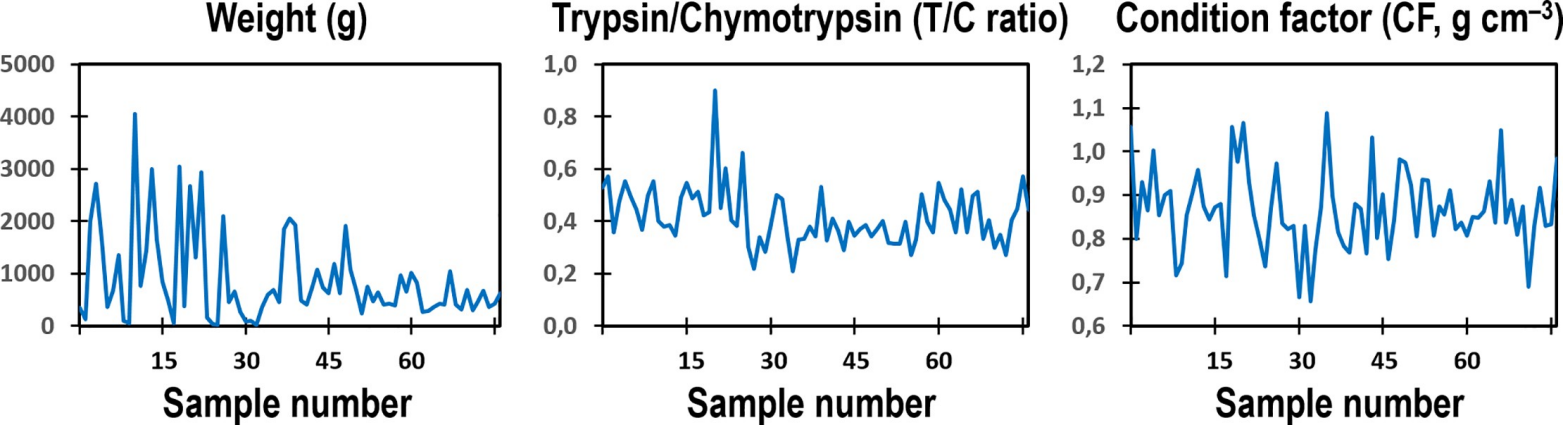
Dataset with weight, pyloric caecal T/C ratio, and CF of wild Northeast Arctic cod at adult stage during spawning migration. Weight, T/C ratio and CF values will be normalized to a range of between –1 and +1 for estimating individual SGR and testing our models. (original data from [12]).

Two datasets were used for testing the computational models. The first dataset, with complete biological factors of weight, T/C ratio, CF and SGR, was from a growth experiment of adult Nile tilapia (*Oreochromis niloticus* L.) with different feeding regimes (Data5: *n* = 31, Fig 2, personally provided by Karun Thongprajukaew, Prince of Songkla University, Thailand). The second dataset was from the unprecedented study of wild Northeast Arctic cod (*Gadus morhua* L.) at adult stage during spawning migration in three studied areas of the Barents Sea [12]. Area A was between North Kanin Bank and Eastern Basin, area B was Kanin Bank, and area C was Central Bank, with average bottom temperatures of 1.61 ^o^C, 3.45 ^o^C, and 1.69 ^o^C, respectively (Fig 3). This dataset included the three important factors (weight, T/C ratio, CF, *n* = 77, Fig 4), and was used for individually estimating the SGR of the cod caught in the three different sea areas with variations in temperatures and food diversity. Such growth estimates could become future common practice for different wild populations in natural ecosystems where the individual fishes, environmental conditions, food availability, and consumption rates under study cannot be controlled.

### Neural computational model

**Fig 5:**
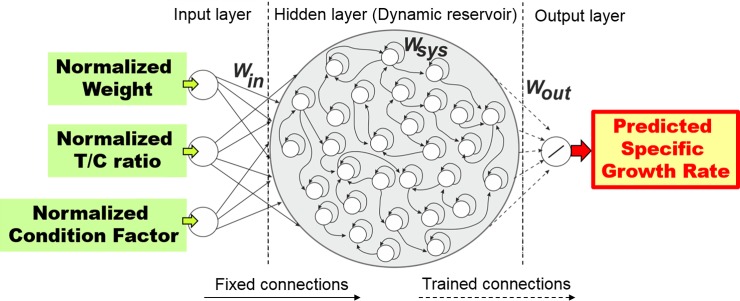
An example of our reservoir-based neural network computational model. In this setup, the input(s) to the reservoir network come from weight, activity ratio of trypsin to chymotrypsin (T/C ratio) in fish pyloric caeca and small intestine, condition factor (CF), or their combinations. The reservoir learns to produce estimated or predicted specific growth rate (SGR) outputs.

The computational model was developed using recurrent neural networks (RNNs) of the reservoir computing (RC) type [19,22]. It is based on the RC framework of Dasgupta *et al*. [22] and the time-series data processing mechanisms developed from the Plan4Act project (funded by FETPROACT-01-2016—FET Proactive under grant agreement no. 732266). Due to the dynamic reservoir, the network with recurrent connections exhibits a wide repertoire of nonlinear activity and temporal memory. These features can be exploited for precise SGR estimation. In this study, the reservoir-based neural network has three layers: an input layer, a dynamic reservoir hidden layer, and an output layer (Fig 5).

The *dynamic reservoir hidden layer* is constructed with *N* hidden recurrent neurons. These neurons have sparsely recurrent connections which are randomly assigned and remain fixed. The recurrent neural activity within the dynamic reservoir varies as a function of its previous activity and the current driving input signal. The discrete time state dynamics of reservoir neurons is given by:

$$x(t+1)=(1-\lambda )x(t)+\lambda f_{sys}(W_{in}u(t+1)+W_{sys}x(t)+b_{o})$$ (3)

$$y(t)=W_{out}x(t)$$ (4)

where ***x****(t)* is the *N* dimensional vector of hidden neural state activations. *N* is the number of hidden neurons defining the network size, and empirically set. To obtain less computational power while maintaining acceptable data estimation with generalization, the chosen network contains *N* = 50 hidden recurrent neurons with a neural parameter (called spectral radius) of 0.9. It should be noted that using a larger network with more hidden recurrent neurons (e.g. 200 hidden neurons) could lead to overfitting and lack of generalization. Furthermore, a larger network typically needs a longer training time and is computationally expensive. Thus, in this study, the network size is kept to 50 hidden neurons.

The *input layer* is constructed with *M* input neurons. The ***u****(t)* is the *M* dimensional vector of input neural state activations. In this study, *M* can be one, two, or three depending on the input data (i.e. weight, T/C ratio, CF, or their combinations).

The *output layer* is constructed with *J* output neurons. The ***y****(t)* is the *J* dimensional vector of output neural state activations. Here *J* is set to one where the output of the network is estimated or predicted SGR. With this setup, more input and output data can be simply applied by adding further input and output neurons.

The *λ* is the network time scale, where 0 < *λ* ≤ 1. The constant bias *b*_*0*_ = 0.001 is applied to the hidden recurrent neurons of the network. The bias term is used in order to provide a small input for the hidden neurons to constantly activate them, thereby maintaining the neurodynamics. In doing so, the network provides better data estimation than without the bias term. ***W***_*in*_ and ***W***_*sys*_ are the input weights (projecting from the input neurons to the hidden recurrent neurons) and the hidden recurrent connection weights of the hidden neurons, respectively. They are set randomly from a uniform distribution of [–0.1, 0.1] and [–1, 1], respectively. The recurrent neurons are updated with a *tanh* nonlinear activation function, *f*_*sys*_ = *tanh(x)* where *x* is the right-hand side of Eq 3 inside the function *f*_*sys*_. The output neuron uses a linear activation function. The output weights ***W***_*out*_ (trained connections, see dashed arrows in Fig 5) are calculated using the recursive least squares (RLS) algorithm [23] at each time step, while the input ***u****(t)* is being fed into the network. The output weights ***W***_*out*_ are calculated such that the overall error is minimized. As a consequence, the network transforms weight, T/C ratio, CF, or their combinations to estimate the SGR precisely. The RLS algorithm is implemented using a fixed forgetting factor (*λ*_*RLS*_ < 1) as follows:

e(t)=d(t)−y(t) (5)

$$K(t)=\frac{p(t-1)x(t)}{\lambda_{RLS}+x^{T}(t)p(t-1)x(t)}$$ (6)

$$\left(t)=\frac{1}{\lambda_{RLS}}[p(t-1)-K(t)x^{T}(t)p(t-1)\right]$$ (7)

$$W_{out}(t)=W_{out}(t-1)+K(t)e(t)$$ (8)

Here, for each input set ***u****(t)*, the reservoir state ***x****(t)* and network output ***y****(t)* are calculated using Eqs 3 and 4. The *e(t)* is the error calculated from the difference between the desired output or target *d(t)* (i.e. SGR) and the generated output. ***K****(t)* is the RLS gain vector and ***p****(t)* is the auto-correlation matrix updated at each time step. The output weights ***W***_*out*_ are initially set to zero. The forgetting factor *λ*_*RLS*_ is set to a value of less than one (i.e. 0.99). The auto-correlation matrix ***p*** is initialized as *p(0)* = ***I****/β*, where ***I*** is the unit matrix and *β* is a small constant (i.e. 10^−4^). Details of all the fixed parameters and initial settings for the reservoir model are summarized in Table 1.

**Table 1:** List of the reservoir-based network parameter settings.

Parameter	Value
Reservoir network size (neurons)	50
Number of output neurons	1
Number of input neurons	1–3
Reservoir neuron bias (*b*_*o*_)	0.001
Network time scale (*λ*)	0.3 or 1.0 depending on dataset
RLS learning constant (*β*)	10^−4^
RLS learning rate (*λ*_*RLS*_)	0.99
Reservoir network sparsity	50%
Input to reservoir sparsity	50%
Reservoir spectral radius	0.9

RLS = the recursive least squares algorithm

The network is trained in an online manner where the output weights ***W***_*out*_ of the network are updated at each input-target pair. To obtain different network output weights for our neural model in this study, we use seven sets of inputs from each fish dataset: Set 1 (only weight), Set 2 (only T/C ratio), Set 3 (only CF), Set 4 (weight and T/C ratio), Set 5 (T/C ratio and CF), Set 6 (weight and CF), Set 7 (weight, T/C ratio, and CF). These input data are normalized using the min–max normalization technique. The technique simply normalizes the input data through division by the data range (max–min), as shown in Eq 9:

Normalizedinputdata=(Inputdata−min)/(max−min) (9)

In doing so, the input range is linearly transformed to the interval [−1, 1]. This interval is used with respect to the range of the *tanh* activation function of the hidden neurons in the network. An example of the setup of the network is shown in Fig 5. Basically, for seven sets of the input combinations for one fish dataset, seven sets of network output weights are obtained. Applying this setup for three fish datasets; i.e. Atlantic salmon at juvenile stage (Data1, Fig 1A) and post-smolt stage (Data2, Fig 1B), and rainbow trout at adult stage (Data4, Fig 1D), we then obtain 21 sets of the network output weights in total, which are stored as our trained computational model for SGR estimation.

## Results

### Biological data for developing the model

**Fig 6:**
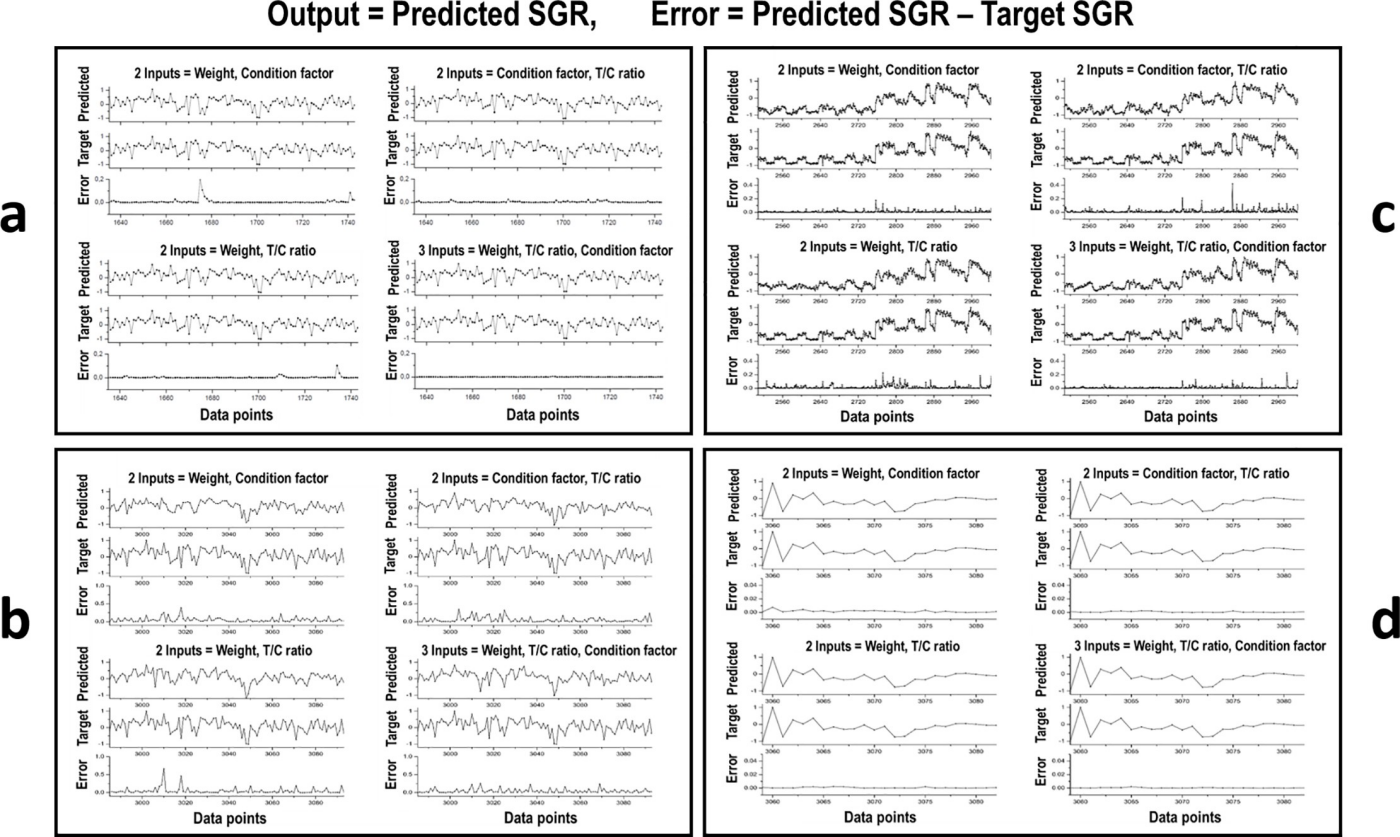
Comparison of predicted specific growth rate (SGR) outputs with the target normalized SGR using different input combinations. Different inputs to the reservoir-based neural network of our computational model were studied, using different reservoir network sizes of hidden neurons. The three-input combination of normalized weight, normalized pyloric caecal activity ratio of trypsin to chymotrypsin (T/C ratio), and normalized condition factor (CF) provided the best results of predicted SGR values with the lowest average error, compared to the two-input combinations. The importance of the T/C ratio input is also illustrated to provide less error in predicted SGR values. (a) Data4: Rainbow trout at adult stage during maturation (Fig 1D) using a large reservoir network size of 200 hidden neurons. The average errors in the three-input combination, and two-input combinations of the weight and T/C ratio, the T/C ratio and CF, and the weight and CF were 0.00047, 0.0038, 0.0039, and 0.0078, respectively. (b) Data4: Rainbow trout at adult stage during maturation (Fig 1D) using a reservoir network size of 50 hidden neurons. (c) Data1: Atlantic salmon at juvenile stage (Fig 1A) using a reservoir network size of 50 hidden neurons. (d) Data2: Atlantic salmon at post-smolt stage (Fig 1B) using a reservoir network size of 50 hidden neurons. For (b), (c), and (d), using 50 hidden neurons, the average errors in the three-input combination and two-input combinations are shown in Table 2.

**Fig 7:**
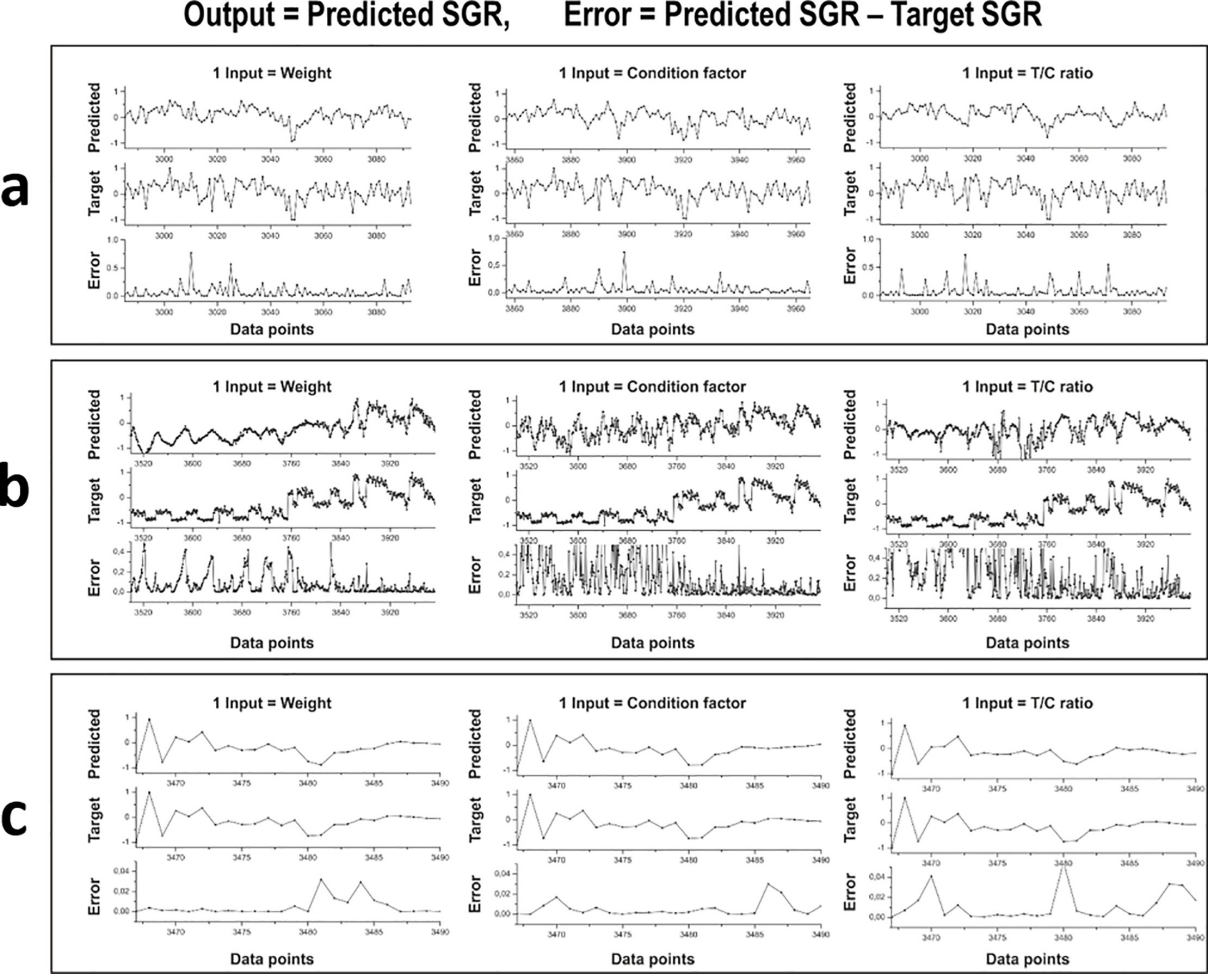
Comparison of predicted specific growth rate (SGR) outputs with the target normalized SGR using different single inputs. Different single inputs (weight, condition factor, or T/C ratio) into the reservoir-based neural network of our computational model were investigated, using a reservoir network size of 50 hidden neurons. (a) Data4: Rainbow trout at adult stage during maturation (Fig 1D). (b) Data1: Atlantic salmon at juvenile stage (Fig 1A). (c) Data2: Atlantic salmon at post-smolt stage (Fig 1B). Single inputs are not suitable because of a large number of errors in SGR estimation. The mean square errors using single inputs are shown in Table 2.

According to the different input combinations of the datasets used for testing the model, the results indicated that the higher the input factor, the lower the SGR estimation error, and the inputs with the T/C ratio provided less error in SGR estimation. The results of different combined inputs (Fig 6), and one factor input with a large number of errors (Fig 7) are illustrated. The mean square errors (MSEs) of the different inputs are shown in Table 2. The three-input combination of weight, T/C ratio, and CF provided the best SGR estimations (see Fig 6 and Table 2). A preliminary study of Data4, using a large reservoir network size of 200 hidden neurons, indicated precise SGR estimations from the three-input combination with the lowest average error of 0.00047 (almost no error), compared to two-input combinations with an average error range of 0.0038–0.0078 (Fig 6A). The studies on Data4 (Fig 6B), Data1 (Fig 6C), and Data2 (Fig 6D), using 50 hidden neurons, showed acceptable predicted SGR values from the three-input combination with an average error range of 0.0004–0.0436 (see Table 2). The use of 50 hidden neurons can reduce the computational time and cost, compared to 200 hidden neurons. Therefore, all trained models for SGR estimation were used with a reservoir network size of 50 hidden neurons and the three-input combination of normalized weight, normalized T/C ratio, and normalized CF.

**Table 2:** Mean square errors (MSEs) for different input combinations (one input, two inputs, or three inputs) of our computational models with a reservoir network size of 50 hidden neurons. The MSE is calculated from the target (or real) SGR and the predicted (or estimated) SGR values shown in Figs 6 and 7.

Fish data	One input			Two inputs			Three inputs
	Weight (W)	T/C ratio (T/C)	Condition factor (CF)	W, T/C	T/C, CF	W, CF	W, T/C, CF
Rainbow trout at adult stage (Fig 1D)	0.0784 ± 0.1239	0.0765 ± 0.1373	0.0601± 0.1002	0.0470 ± 0.0871	0.0523 ± 0.0717	0.0514 ± 0.0623	0.0436 ± 0.0546
Atlantic salmon at juvenile stage (Fig 1A)	0.0779 ± 0.1032	0.2604 ± 0.3195	0.1749 ± 0.2385	0.0156 ± 0.0275	0.0156 ± 0.0296	0.0089 ± 0.0160	0.0088 ± 0.0189
Atlantic salmon at post-smolt stage (Fig 1B)	0.0050 ± 0.0088	0.0114 ± 0.0147	0.0053 ± 0.0075	0.0012 ± 0.0021	0.0007 ± 0.0008	0.0017 ± 0.0017	0.0004 ± 0.0005

For model evaluation, 15% of each dataset was tested from the trained model using 85% of the entire dataset. In addition, the 15% of each dataset was also tested from the trained models with combinations of different datasets. The results of estimated SGR outputs were compared with the real SGR values to obtain the mean squared error (MSE), as shown in Table 3. Surprisingly, the outputs of estimated SGR values were within an acceptable range (small MSE), regardless of the trained models. This means that a combination of different datasets from different species, with variations in age and growth stages, could be used as a common trained model for generality of growth estimation of any species and size.

**Table 3:** Growth estimates of weight specific growth rate (SGR) in % per day (mean ± SD) for different datasets from different species and at different growth stages, with three inputs of normalized weight, normalized activity ratio of trypsin to chymotrypsin (T/C ratio), and normalized condition factor (CF), using different trained models from each dataset and combinations of different datasets. The values in parentheses are mean squared error (MSE) calculated from real SGR and estimated SGR values. The network time scale *λ* = 0.3 for the models from the same dataset, and *λ* = 1.0 for the other models (see Table 1).

Trained model	Growth estimate tests and MSE from 15% of known selected data (% day^–1^)					
	Real SGR value	Trained model from the same Data-set	Trained model Data1 + Data2	Trained model Data2 + Data4	Trained model Data1 + Data4	Trained model Data1 + Data2 + Data4
Data1: Atlantic salmon at juvenile stage (499 data points, see Fig 1A)	2.0616 ± 0.4538	2.3481 ± 0.4538 (0.1672±0.1676)	2.3569 ± 0.4936 (0.2594±0.3005)	2.1410 ± 0.2483 (0.1655±0.1286)	2.2551 ± 0.3691 (0.1729±0.1950)	2.2422 ± 0.3602 (0.1841±0.2429)
Data2: Atlantic salmon at post-smolt stage (24 data points, see Fig 1B)	0.8520 ± 0.0377	0.7625 ± 0.1745 (0.0386±0.0483)	0.7001 ± 0.4078 (0.1437±0.1625)	0.9334 ± 0.1331 (0.0285±0.0457)	0.7625 ± 0.2978 (0.0951±0.1228)	0.9452 ± 0.2507 (0.0527±0.0345)
Data4: Rainbow trout at adult stage (109 data points, see Fig 1D)	0.4282 ± 0.1293	0.4289 ± 0.0662 (0.0162±0.0180)	0.4736 ± 0.1329 (0.0281±0.0393)	0.4149 ± 0.0905 (0.0141±0.0185)	0.4667 ± 0.1138 (0.0191±0.0214)	0.4931 ± 0.1009 (0.0186±0.0213)

### Biological data for testing the model

**Fig 8:**
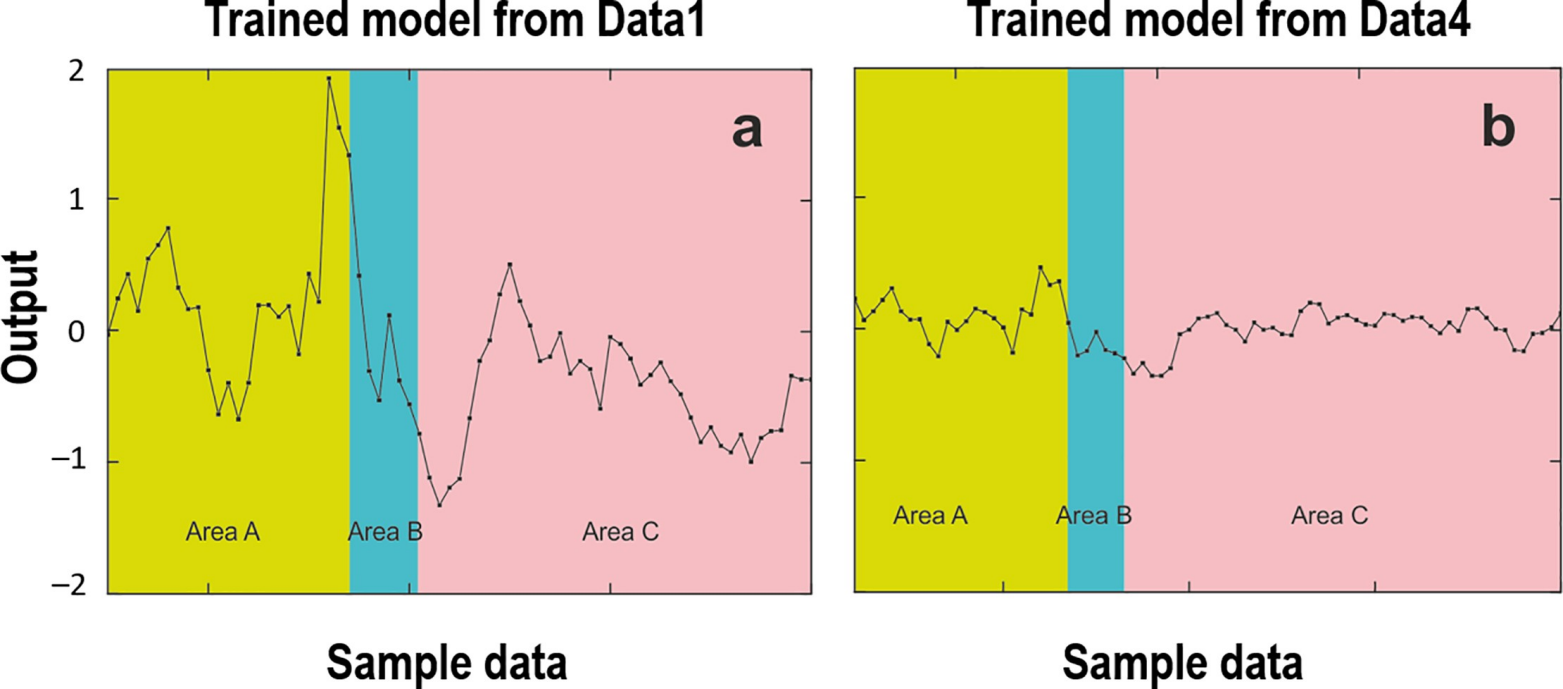
Weight specific growth rate (SGR) predictions (outputs) of wild Northeast Arctic cod at adult stage during spawning migration in three different Barents Sea areas (see Figs 3 and 4). A comparison was made using two trained weight-parameter sets for the models of SGR estimates in (a) Data1: Atlantic salmon at juvenile stage (Fig 1A), and (b) Data4: rainbow trout at adult stage (Fig 1D), to predict the SGR of wild Northeast Arctic cod at adult stage. To test the predictions, we used the normalized weight, normalized pyloric caecal activity ratio of trypsin to chymotrypsin (T/C ratio) and normalized condition factor (CF) of the wild Northeast Arctic cod at adult stage as the model inputs (see Figs 4 and 5). The models generate normalized SGR predictions for the wild Northeast Arctic cod at adult stage in area A (between North Kanin Bank and Eastern Basin), area B (Kanin Bank), and area C (Central Bank).

The dataset of wild Northeast Arctic cod from three Barents Sea areas (Figs 3 and 4) was used for testing different trained models. Comparisons were made to show the SGR estimates from the network output weights of two trained models (Fig 8), based on the normalized datasets with three inputs of Atlantic salmon at juvenile stage (Fig 1A) and rainbow trout at adult stage (Fig 1D), representing different species at different growth stages. The estimated SGR patterns (Fig 8A and 8B) from the two tests were similar, but with different amplitudes of absolute values, showing higher output values with the trained model from younger fish of Atlantic salmon at juvenile stage compared to rainbow trout at adult stage, as also shown in Table 4. This is due to the fact that younger fish usually have a higher growth rate than older fish. It should be noted that the output values (estimated SGR values) of the trained models were converted to the estimated real SGR values by scaling them linearly based on the maximum and minimum values of all real SGR values of the input data. For example, the output values of the trained model from Data1+Data2+Data3+Data4+Data5 were converted to the estimated real SGR values by scaling them based on the maximum and minimum values of all real SGR values of the combined datasets of Data1, Data2, Data3, Data4, and Data5. The results in Fig 8 and Table 4 indicate a possibility of using any trained models for comparison of SGR estimates, regardless of age, growth stage and species. However, a trained model with a combination of more datasets from different species with variations in size will provide more correct absolute values of SGR estimations (see Table 4). All outputs from different trained models of each dataset and their combinations confirmed the ranking of SGR estimated values for the wild Northeast Arctic cod in the three Barents Sea areas, as A > C > B, regardless of the trained models (Table 4). The results provided a consistent ranking of SGR estimated values among the three areas but with different amplitudes of absolute values, depending on the majority of the combined datasets. At this point, the correct absolute values of SGR estimates for the cod in each Barents Sea area should be from the trained model of the combination of Data 1–4 (see Fig 1), for temperate species.

**Table 4:** Growth estimates of weight specific growth rate (SGR) in % per day (mean ± SD) of Nile tilapia (see Fig 2) and wild Northeast Arctic cod in three different areas of the Barents Sea (see Figs 3 and 4) using different trained models from each dataset and combinations of different datasets with three inputs of normalized weight, normalized activity ratio of trypsin to chymotrypsin (T/C ratio), and normalized condition factor (CF). The values in parentheses are mean squared error (MSE) calculated from the real and estimated SGR values. The network time scale *λ* = 1.0 for all models (see Table 1).

Trained model	Nile tilapia		Wild Northeast Arctic cod in the Barents Sea		
	Real SGR value	Estimated SGR value	Estimated SGR in area A	Estimated SGR in area B	Estimated SGR in area C
Data1 (see output profile in Fig 8A)	2.81 ± 0.39	2.59 ± 0.36 (0.1505±0.2001)	2.18 ± 0.84	1.11 ± 0.51	1.17 ± 0.50
Data4 (see output profile in Fig 8B)	2.81 ± 0.39	2.55 ± 0.33 (0.2006±0.2233)	0.45 ± 0.07	0.32 ± 0.04	0.41 ± 0.06
Data1 + Data2	2.81 ± 0.39	2.61 ± 0.36 (0.1038±0.1251)	1.56 ± 0.41	0.83 ± 0.19	1.02 ± 0.40
Data2 + Data4	2.81 ± 0.39	2.65 ± 0.28 (0.1660±0.2177)	0.74 ± 0.24	0.58 ± 0.14	0.64 ± 0.13
Data1 + Data2 + Data4	2.81 ± 0.39	2.64 ± 0.33 (0.1564±0.1944)	1.41 ± 0.52	0.90 ± 0.42	1.10 ± 0.39
Data1 + Data2 + Data3 + Data4	2.81 ± 0.39	2.70 ± 0.28 (0.0968±0.1344)	1.42 ± 0.63	0.68 ± 0.45	1.28 ± 0.38
Data1 + Data2 + Data3 + Data4 + Data5	2.81 ± 0.39	2.73 ± 0.28 (0.0313±0.0249)	1.57 ± 0.72	1.04 ± 0.49	1.43 ± 0.35

Data1: Juvenile Atlantic salmon (499 data points, see Fig 1A), Data2: Post-smolt Atlantic salmon (24 data points, see Fig 1B)

Data3: Adult Atlantic salmon (79 data points, see Fig 1C), Data4: Adult rainbow trout (109 data points, see Fig 1D)

Data5: Nile tilapia (31 data points, see Fig 2)

The dataset of a tropical species, Nile tilapia, with known SGR (Fig 2) was also used for testing different trained models in the same way as the dataset from the wild Northeast Arctic cod (Table 4). The outputs of the mean SGR estimated values of Nile tilapia showed that the higher the combination of the datasets in the trained model, the lower the mean square error of SGR estimation (Table 4). In this case, the model from Data1+Data2+Data3+Data4+Data5 is the best SGR estimation for Nile tilapia because of the lowest mean square error (see Table 4). This is probably due to the inclusion of the dataset from Nile tilapia (Data5) into the model. In addition, it also indicates the possibility of using trained models of three-input combinations with a high combination of datasets from temperate species (Data 1–4) for SGR estimation of tropical species (see Table 4).

## Discussion

At this point, the neural computational model *GrowthEstimate* has been developed with a combination of four datasets from salmonids of different sizes, containing key biological factors of weight (g), digestive efficiency (T/C ratio), protein growth efficiency through the condition factor (CF, 100 × g cm^–3^), and weight specific growth rate (SGR, % day^–1^) (Fig 1). Although developed from temperate species, it can also estimate the SGR of tropical species (see Table 4).

Growth models in the stock assessment of wild marine populations can strongly influence the estimated biomass, affecting the conclusion of stock status and exploitation level, and age-length data for growth estimates can be biased if the stocks have potential regional differences in growth, and age and sex-specific movements [24]. Most growth models consider the values for the parameters of a size-transition matrix, and the approaches used to model growth have been reviewed with comments on uncertainty and biases, due to a number of complicated and interacting issues that are often not explicitly recognized with unknown consequences for an assessment [25,26]. Growth rate variations among individual fishes may be persistent or transient, whereas the transient variation accounts for up to half of the total variability [27]. According to our knowledge, those unrecognized transient factors (affecting differences in the yearly growth rate) could probably represent the individual variations in food utilization efficiency, genetically affected and influenced by the quality and availability levels of food and changes in environmental conditions [1–4,12]. Since the digestive efficiency T/C ratio and protein growth efficiency through CF are related to growth potential [1–4], the inclusion of these biological factors in actual animal responses, together with weight, for training our *GrowthEstimate* model, will provide precise SGR estimates for the animals without any assumptions. Collection of the extraordinary biochemical data of the digestive efficiency T/C ratio coupled with normal fishery data (e.g. weight and length) is needed for improving the stock assessment process.

Genetic variations in food digestion and utilization for optimal growth could be indirectly studied through the specific activities of trypsin (T) and chymotrypsin (C) in the pyloric caeca, small intestine, or digestive gland (depending on the species studied) with food content (as the two active proteases are secreted into the lumen) [1–4]. The enzymatic studies provide the calculated T/C ratio values of individual fishes, not only for use in our neural computation model for precisely estimating individual specific growth rates, but also for predicting future growth potential as the T/C ratio could predict growth over a period of 1–2 months [9,11]. The levels of T/C ratio values increase with growth potential, and younger animals have higher growth potential than older ones. This has been clearly observed during the molting period and metamorphosis, respectively, in the blue swimming crab *Portunus pelagicus* L. [14]. Therefore, the age of the growth stage specified through the animal body weight at sampling is important for growth rate estimation. However, growth performance quality is dependent on protein deposition in the body and white muscle. This is because a higher dietary protein level could result in a higher protein deposition and reduced condition factor, due to protein deposition being more associated with body length than body weight [13]. Therefore, the condition factor is the important indirect indicator for protein growth efficiency.

**Fig 9:**
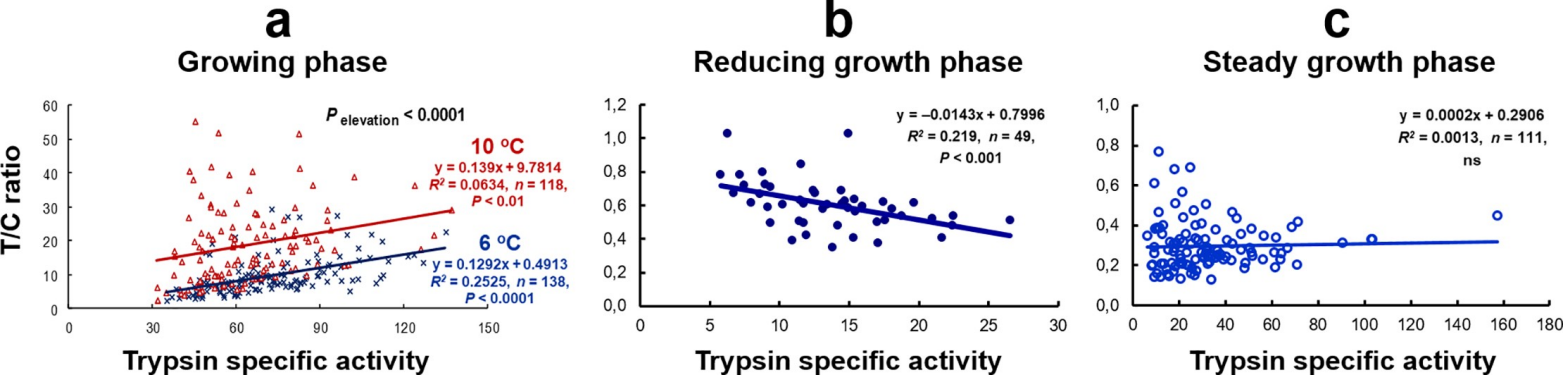
Growth status studies by the relationship between trypsin specific activity and the activity ratio of trypsin to chymotrypsin (T/C ratio) in the pyloric caeca with food content. (a) *During the growing phase* in Atlantic salmon parr reared at 6 ^o^C (×) and 10 ^o^C (Δ) (adapted from [9], with permission from Springer Corp.). (b) *During the reducing growth phase* in Atlantic salmon of about 1 kg (data adapted from [28], with permission from John Wiley and Sons, Inc.). (c) *During the steady growth phase* in maturing rainbow trout (data adapted from [13], with permission from Hindawi Publishing Corp.). The enzyme specific activities of trypsin and chymotrypsin are expressed as μmol *p*-nitroaniline produced h^–1^ mg protein^–1^. ns, not significant.

In addition, the slope T/C ratio, obtained from a regression between specific activities of trypsin (*y*-axis) and chymotrypsin (*x*-axis) of the individuals in each population, could indicate a comparable growth rate at sampling between different populations [1–4]. Moreover, a regression slope between T/C ratio values (*y*-axis) and trypsin specific activities (*x*-axis) of the individuals in a population could indicate the growth status of the population at sampling, whether they are at a growing phase with positive regression (Fig 9A), a reducing growth phase with negative regression (Fig 9B), or a steady growth phase with no relationship (Fig 9C) [1–4]. A population with a regression showing a higher slope or elevation (see Fig 9A) than others indicates a higher growth rate [9,11,12]. The implementation of these factors for studying growth of living resources in natural marine ecosystem is important, and has been performed on wild Northeast Arctic cod in the Barents Sea, making a difference to marine research [12]. The higher average SGR estimates of the wild Northeast Arctic cod from area A compared to area C (Fig 8 and Table 4) corresponded with the higher T/C ratio values and elevation of slope T/C ratio observed in these fish [12]. In addition, the lowest estimated levels of average SGR for the wild Northeast Arctic cod from area B (Fig 8 and Table 4) are also reasonable, because the cod in area B with higher temperatures and food variety showed a higher maturation rate than those in areas A and C [12], as the fish with higher maturation rate will reduce more SGR [13]. Moreover, a higher T/C ratio value of trypsin-like to chymotrypsin-like activity ratio in oocytes also showed a higher oocyte growth, indicating a higher female maturation rate. This has been observed in the cod from area B, despite the lower specific activities of the two oocyte proteases, compared to those from areas A and C [12].

Our neural computational model *GrowthEstimate* has also been developed using recurrent neural networks of the reservoir computing type [19,22]. In general, artificial neural networks (ANNs) have been widely used in many applications such as image processing [29], weather forecasting [30], medicine [31], biology [32–36], robotics [37], and the nonlinear modeling of temporal data [19,31,38,39]. They have also been applied to fish data mainly for species identification [40–45], prediction [46,47], and classification [48,49]. Additionally, they have been used as a forecasting tool for fisheries [50]. In contrast to these applications, reservoir computing type recurrent neural networks are employed in this study as our computational model for estimating the nonlinear time-series data of different fish stages and species. As a result, the model can precisely estimate SGRs (% day^–1^) particularly based on the combination of weight (g), digestive efficiency (T/C ratio), and protein growth efficiency through the condition factor (CF, 100 × g cm^–3^) of the fish data. The three-factor-input has provided the least SGR estimation errors (see Fig 6 and Table 2). This neural computational model with the combined inputs of normalized weight, normalized digestive efficiency (T/C ratio), and protein growth efficiency through normalized condition factor, could precisely estimate the comparable SGRs of living resources, especially in natural marine ecosystems. A combination of different datasets, with similar data points of different species with variations in age and growth stages, will be a common trained model for growth estimates in any species of different sizes.

At this point, it could be concluded that the model with three inputs of combined datasets of Data1, Data2, Data3, and Data4 (Fig 1) is the most suitable *GrowthEstimate* model for temperate species, and probably also for tropical species (see Fig 6 and Table 4). The model can be improved by increasing the data points from both temperate and tropical species of different sizes during the later stages of our development, to produce a common model for generality. This is possible because each species will have specific weight and CF values from which the model can learn, and use the T/C ratio to include in growth estimation. Co-operations are needed for the biodata collections of different species from different climate zones for generality of the *GrowthEstimate* model for future use.

## Conclusions

The neural computational model *GrowthEstimate* is developed using the combined inputs of normalized weight (g), normalized digestive efficiency (T/C ratio), and protein growth efficiency through the normalized condition factor (CF, 100 × g cm^–3^). The model could precisely estimate the weight specific growth rate (SGR, % day^–1^) of living resources, especially in natural marine and freshwater ecosystems where food availability, consumption rates, and growth rates are unknown. However, the trained model should be improved with additional datasets with similar data points from different species at different development stages. Determinations of the activities of trypsin and chymotrypsin in the pyloric caeca and small intestine for the T/C ratio study, including other biochemical techniques for studying the performance qualities of growth and maturation in aquatic living resources, are described in [4,28]. The advantages of simultaneously using different biochemical techniques developed by Rungruangsak-Torrissen and her research team are described [10,12,13,28,51], and summarized in [1–4]. The importance of the specific activity levels of trypsin and chymotrypsin for fish growth is similar to the importance of the levels of acceleration and braking capacity for car speed, respectively. A higher acceleration (trypsin specific activity) is needed to increase car speed (fish growth), and a higher braking capacity (chymotrypsin specific activity) is necessary for stopping a car (fish) at a higher speed (higher growth) [4,13,51].

This study provides a research revelation resulted from an excellent collaboration between different research fields in life science (nutritional biochemistry) and computational neuroscience. Insights from the breakthrough of the biological studies [1–4,7] have provided the unique key biological factors, indicating the growth performance quality of living resources through the digestive efficiency T/C ratio and condition factor (as protein growth efficiency). These factors are applicable for practical studies directly in natural ecosystems where individuals in populations, environmental conditions, food availability and consumption rates cannot be controlled. The newly developed neural computational model *GrowthEstimate* in this study, using reservoir-based recurrent neural networks, is a promising technique for precise and comparable growth estimates of living resources. The *GrowthEstimate* software, using the weight, T/C ratio, and CF, as its inputs, and providing the estimation of SGR as its output, can be a valuable tool for fishery scientists and other researchers in studying the growth rates of individual fishes, without knowing about food availability, consumption rates, and their genetics. Thus, it can be used as a powerful tool for future food utilization and growth studies, especially in natural ecosystems. Such studies will improve our knowledge and make a difference in studying biochemical effects of environmental impact and climate change on the survival and growth of living resources.
