# Correction: Neural computational model *GrowthEstimate*: A model for studying living resources through digestive efficiency

**DOI:** 10.1371/journal.pone.0223740

**Published:** 2019-10-07

**Authors:** The PLOS ONE Staff

In the Author Contributions, Poramate Manoonpong (PM) should be listed as one of the persons who acquired funding.

There is an error in affiliation 1 for author Krisna Rungruangsak-Torrissen. The correct affiliation 1 is: Institute of Marine Research, Ecosystem Processes Research Group, Matre Research Station, Matredal, Norway. The publisher apologizes for the error.

There are errors in references 1, 2, and 3, The correct reference 1 is: Rungruangsak-Torrissen K. Trypsin and its implementations for growth, maturation, and dietary quality assessment. In: Weaver K, Kelley C, editors. Trypsin: Structure, Biosynthesis and Functions. New York: Nova Science Publishers Inc; 2012. pp. 1–59. http://www.novapublishers.org/catalog/product_info.php?products_id=38114

The correct reference 2 is: Rungruangsak-Torrissen K. Atlantic Salmon, Salmo salar L.: Genetic Variations in Protein Metabolism and Growth. In: Woo PTK, Noakes DJ, editors. Salmon: Biology, Ecological Impacts and Economical Importance. New York: Nova Science Publishers Inc; 2014. pp. 85–120. https://novapublishers.com/wp-content/uploads/2019/05/978-1-63117-570-1_ch6.pdf

The correct reference 3 is: Rungruangsak-Torrissen K. Atlantic Salmon, Salmo salar L.: Food Utilization, Protein Growth Efficiency and Maturation. In: Woo PTK, Noakes DJ, editors. Salmon: Biology, Ecological Impacts and Economical Importance. New York: Nova Science Publishers Inc; 2014. pp. 121–154. https://novapublishers.com/wp-content/uploads/2019/05/978-1-63117-570-1_ch7.pdf. The publisher apologizes for the errors.
